# Therapeutic efficacy of direct oral anticoagulants and vitamin K antagonists for left ventricular thrombus: Systematic review and meta-analysis

**DOI:** 10.1371/journal.pone.0255280

**Published:** 2021-07-26

**Authors:** Tetsuji Kitano, Yosuke Nabeshima, Masaharu Kataoka, Masaaki Takeuchi

**Affiliations:** 1 Department of Cardiology and Nephrology, Wakamatsu Hospital of the University of Occupational and Environmental Health, Kitakyushu, Japan; 2 Second Department of Internal Medicine, University of Occupational and Environmental Health, School of Medicine, Kitakyushu, Japan; 3 Department of Laboratory and Transfusion Medicine, University of Occupational and Environmental Health Hospital, Kitakyushu, Japan; Leiden University Medical Center, NETHERLANDS

## Abstract

**Background:**

Although several meta-analyses have compared efficacies of vitamin K antagonists (VKAs) and direct oral anticoagulants (DOACs) for treatment of left ventricular thrombus (LVT), those meta-analyses included no single-arm studies.

**Methods and results:**

PubMed, Scopus, and the Cochrane Library databases were searched for articles investigating *thrombus resolution*, *stroke*, *any thromboembolis*m, *major bleeding*, *any bleeding*, or *all-cause death* in LVT treated with VKAs or DOACs, and single-class meta-analyses were also included (PROSPERO database, CRD42021230849). Event rates were pooled using a random effects model. Meta-regression analysis was performed to explore factors that may influence outcomes. 2,612 patients from 23 articles were included (VKAs: 2,004, DOACs: 608). There were no significant differences between VKAs and DOACs in the frequency of *thrombus resolution* (VKAs: 0.75 [95% confidence interval; 0.67 to 0.81], DOACs: 0.75 [0.67 to 0.82]), *stroke* (VKAs: 0.06 [0.04 to 0.10], DOACs: 0.02 [0.01 to 0.01]), *any thromboembolism* (VKAs: 0.08 [0.05 to 0.13], DOACs: 0.03 [0.01 to 0.10]), *major bleeding* (VKAs: 0.06 [0.04 to 0.09], DOACs: 0.03 [0.01 to 0.06]), *any bleeding* (VKAs: 0.08 [0.05 to 0.12], DOACs: 0.08 [0.06 to 0.10]), and *all-cause death* (VKAs: 0.07 [0.04 to 0.13], DOACs: 0.09 [0.05 to 0.16]). Meta-regression analysis revealed that increased duration of follow-up was associated with lower-rates of *stroke* (estimate: -0.040, p = 0.0495) with VKAs, but not with DOACs. There was significant publication bias for *thrombus resolution*, *stroke*, *any thromboembolism*, *any bleeding*, and *all-cause death*.

**Conclusions:**

Efficacy and adverse outcomes of therapy with DOACs and VKAs do not differ. Randomized controlled trials are needed to determine the optimal anticoagulant strategy.

## Introduction

Left ventricular thrombus (LVT) present serious complications in patients with stagnant left ventricular (LV) blood flow, patients who have suffered a post-myocardial infarction or heart failure (HF) with severely reduced LV systolic function. With timely and successful percutaneous coronary intervention (PCI) therapy, the incidence of LVT after myocardial infarction (MI) has significantly decreased [1–3]. On the other hand, with aging societies, the prevalence of HF is rapidly increasing [4], and the incidence of LVT associated with impaired LV systolic function may also increase.

For treatment of LVT, European Society of Cardiology guidelines for the management of acute myocardial infarction in patients with ST-segment elevation recommend the use of oral anticoagulants up to 6 months, and American College of Cardiology/American Heart Association guidelines state that anticoagulant therapy with a vitamin K antagonist (VKA) is reasonable for patients with ST-segment elevation MI and asymptomatic LVT [5, 6]. However, VKAs have several disadvantages, including slow onset of action, narrow therapeutic range, the need to monitor the blood international normalized ratio (INR), the necessity of dietary restrictions, and the risk of multiple drug interactions [7]. In contrast, direct oral anticoagulants (DOACs) have fewer drug interactions and are more convenient than VKAs. Currently, DOACs are approved worldwide for treatment of both non-valvular atrial fibrillation (AF) and venous thromboembolism (VTE). In addition, in randomized controlled trials, DOACs have demonstrated some advantages over VKAs for a variety of thromboses, including acute pulmonary thromboembolism [8–10], cancer-related VTE [11–13], non-valvular AF with coronary artery disease [14], and AF with bioprosthetic mitral valves [15]. Although there have been several cases reports and case series on use of DOACs for treatment of LVT, only one randomized controlled trial (RCT) has been published regarding the comparative efficacy and safety of VKAs and DOACs [16].

Recently, several meta-analysis articles have been published comparing VKAs and DOACs in treatment of LVT [17–24]. However, these meta-analyses included only direct comparative studies between VKAs and DOACs, and did not include single-arm studies. The failure to enroll studies that examined only one of the two compound classes could potentially lead to selection bias. Also, some previous publications included studies in abstract form that have not been formally peer-reviewed, and only limited information about these studies is available.

Accordingly, a systematic review and meta-analysis were conducted not only of articles directly comparing efficacy and safety of VKAs and DOACs for treatment of LVT, but also of articles investigating efficacy and safety of either VKAs or DOACs, for therapeutic effects on LVT.

## Materials and methods

### Search strategy

Following Preferred Reporting Items for Systematic Reviews and Meta-Analyses (PRISMA) guidelines, a systematic review and meta-analysis were conducted [25]. PubMed, Scopus, and the Cochrane library electronic databases were searched on May 28, 2021 for articles that investigated the impact of VKAs and/or DOACs on treatment of LVT involving *thrombus resolution*, *stroke*, *any thromboembolism*, *major bleeding*, *any bleeding* and *all-cause death*. Search terms used were *anticoagulant/vitamin K antagonist/warfarin*, *left ventricular/left ventricle/intraventricular*, and *thrombus/thrombi*. Reference lists of all eligible studies were checked for additional citations. Search strategies used are shown in S1 Table. Since DOACs were first released on the global market in 2009, articles that investigated outcomes of VKA and/or DOAC treatment from January 1, 2009 to May 28, 2021 were searched. This study was registered in the PROSPERO database (CRD42021230849).

### Article selection

Articles that met the following criteria were included: (1) patients with LVT who were treated with VKAs or DOACs; (2) articles reporting at least one of the following outcomes: *thrombus resolution*, *stroke*, *any thromboembolism*, *major bleeding*, *any bleeding*, or *all-cause death*; (3) articles reporting information on VKA or DOAC treatment, including numbers of patients, events, and follow-up time; (4) articles on human adults; (5) articles published after 2009. Articles that met the following criteria were excluded: (1) articles that included intracardiac thrombus only in locations other than the left ventricle; (2) articles that included only patients <20 years old or animals; (3) articles that included fewer than 10 eligible patients; (4) case reports or series; (5) abstracts. Two authors (TK and YN) independently reviewed search results and included only articles that were consistent with inclusion and exclusion criteria. Disagreements were resolved either by discussing them or by asking another author (MT) for his opinion.

### Data extraction

Collected data were (1) first author’s name, (2) year of publication, (3) number of study patients, (4) number of events, (5) mean or median age, (6) gender distribution, (7) type of treatment, (8) incidence of events, including *thrombus resolution*, *stroke*, *any embolism*, *major bleeding*, *any bleeding*, *all-cause death*, (9) time to event, (10) type of DOACs (if stated), (11) etiology of heart diseases, and (12) risk factors.

### Statistical analysis

Statistical analyses were conducted using R version 4.0.3 (The R Foundation for Statistical Computing, Vienna, Austria). Continuous data were expressed as means ± SD or medians and interquartile ranges, according to the data distribution, which was determined using the Shapiro-Wilk test. Categorical variables were expressed as absolute numbers or percentages. A single proportion meta-analysis was performed using the “metaprop” with “meta” package, and event rates were pooled using a random effects model to obtain cumulative incidence estimates and 95% confidence intervals (CIs). Subgroup analysis was conducted using the “dmetar” package, and tests for subgroup differences were determined using a mixed/fixed-effects (plural) model. Meta-regression analysis was performed to determine the effect of subgroup and follow-up duration on the outcome. Sensitivity analysis was conducted to assess the influence of outliers on the overall estimate using the “find.outlier” with “dmetar” package. Publication bias was assessed visually based on the distribution of studies, using funnel plots and Egger’s test [26]. For Egger’s test, a p-value < 0.1 was considered significant. The trim and fill method was used to estimate the impact of studies that may have been missing in the meta-analysis [27]. Heterogeneity among studies was assessed using the I^2^ statistic. A two-tailed P value < 0.05 was considered statistically significant. Quality assessment of articles was performed using the Newcastle-Ottawa Assessment Scale (NOS) for non-randomized studies in meta-analysis [28].

## Results

**Fig 1:**
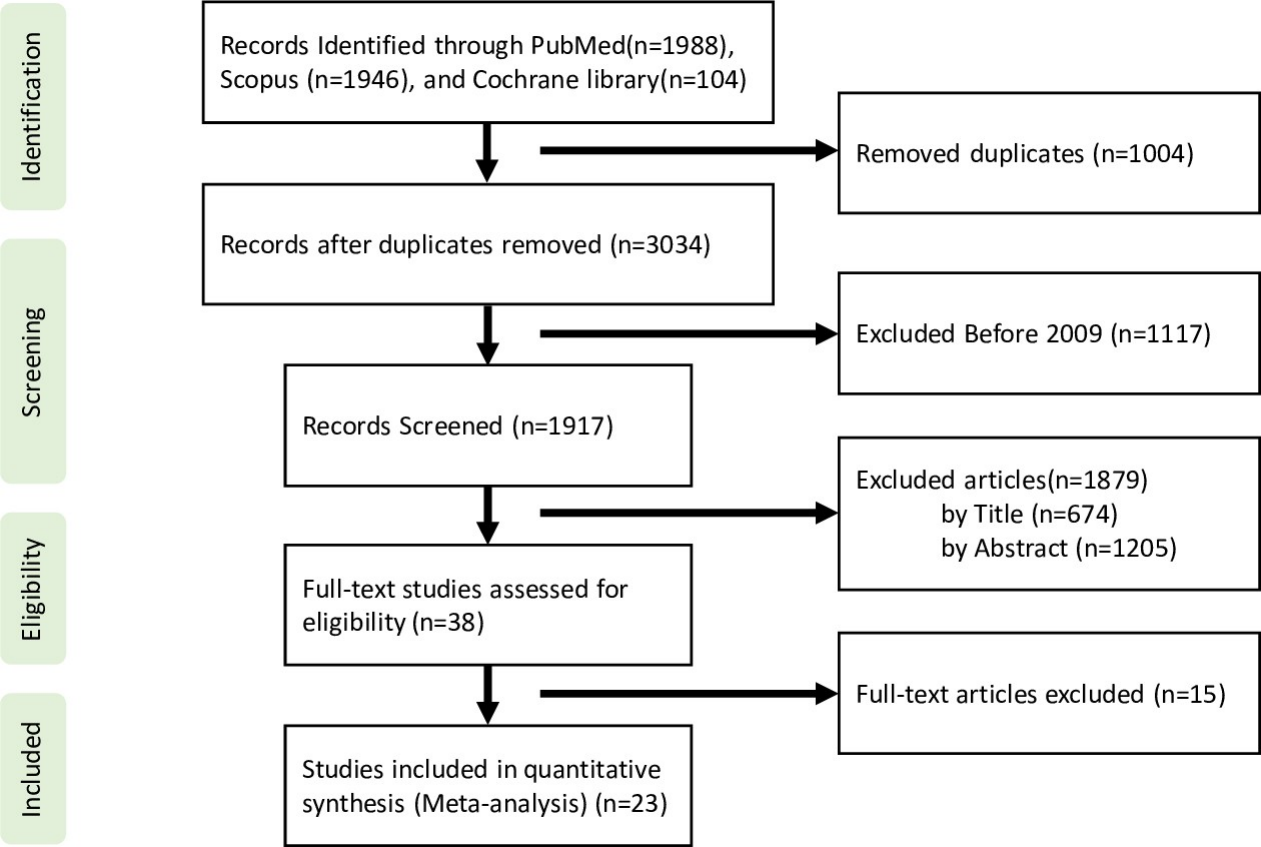
PRISM flowchart of article selection. The systematic review and meta-analysis were conducted according to guidelines recommended by PRISMA.

Articles were selected based on PRISMA guidelines (Fig 1). In total, 4,038 articles were retrieved from the three databases and other sources, of which 1,004 duplicates were excluded. An additional 1,117 were excluded because they were published before 2009, and 1,879 were not included because their titles or abstracts did not fulfill the inclusion criteria, leaving 38 articles for full text evaluation. Fifteen articles that also met one or more exclusion criteria after reading the full text were also eliminated, such that 23 articles were included in this meta-analysis [16, 29–50]. A total of 2,612 patients, 2,004 treated with VKAs and 608 treated with DOACs, were included in this study. S2 Table shows the main characteristics of the study population. Table 1 presents a comparison of clinical characteristics between treatment groups.

**Table 1:** Comparison of clinical characteristics between treatment groups.

Parameters	VKAs (n = 2004)		DOACs (n = 608)		P value
	Number	Measurements	Number	Measurements	
Age	1575	61 ± 15	566	59 ± 16	0.008
Male	1798	1348 (75%)	602	446 (74%)	0.665
HT	910	507 (56%)	309	187 (61%)	0.144
DM	1029	351 (34%)	355	124 (35%)	0.796
DL	666	306 (46%)	240	121 (50%)	0.258
CKD	1059	318 (30%)	257	65 (25%)	0.146
CAD	1194	651 (55%)	235	110 (47%)	0.032
AFib	1523	471 (31%)	439	164 (37%)	0.013
Smoking	464	197 (42%)	151	55 (36%)	0.216

Data are expressed as numbers or means ± standard deviations.

AFib, atrial fibrillation; CAD, coronary artery disease; CKD, chronic kidney disease; DL, dyslipidemia; DM, diabetes mellitus; DOAC, direct oral anticoagulant; HT, hypertension; VKA, vitamin k antagonist.

### Left ventricular thrombus resolution

**Fig 2:**
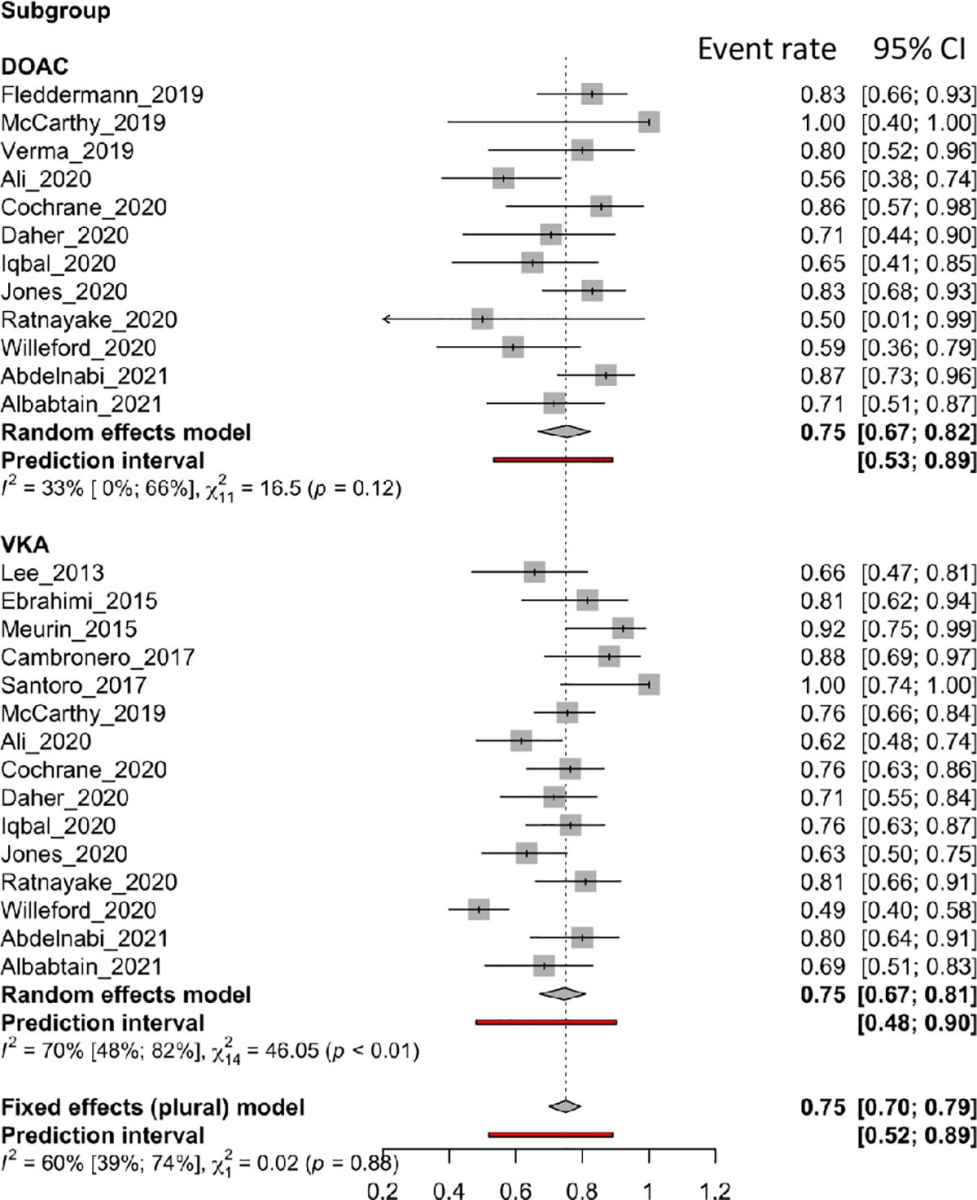
Forest plot of the proportion of left ventricular thrombus resolution rate for DOACs and VKAs therapy.

Among the 23 articles, 17 reported LV *thrombus resolution*, including 15 in the VKA treatment group and 12 in the DOAC group. Fig 2 shows forest plots of the *thrombus resolution* rate with VKAs and DOACs. The pooled analysis presented a similar rate and 95% CI of LV *thrombus resolution* between the two groups (VKAs: 0.75 [95% CI: 0.67 to 0.81]; DOACs: 0.75 [0.67 to 0.82]). Subgroup results showed no differences between the two compound classes (p = 0.8799). Substantial heterogeneity was found in the VKA group (VKAs, I^2^: 70%; DOACs, I^2^: 33%).

### Stroke

**Fig 3:**
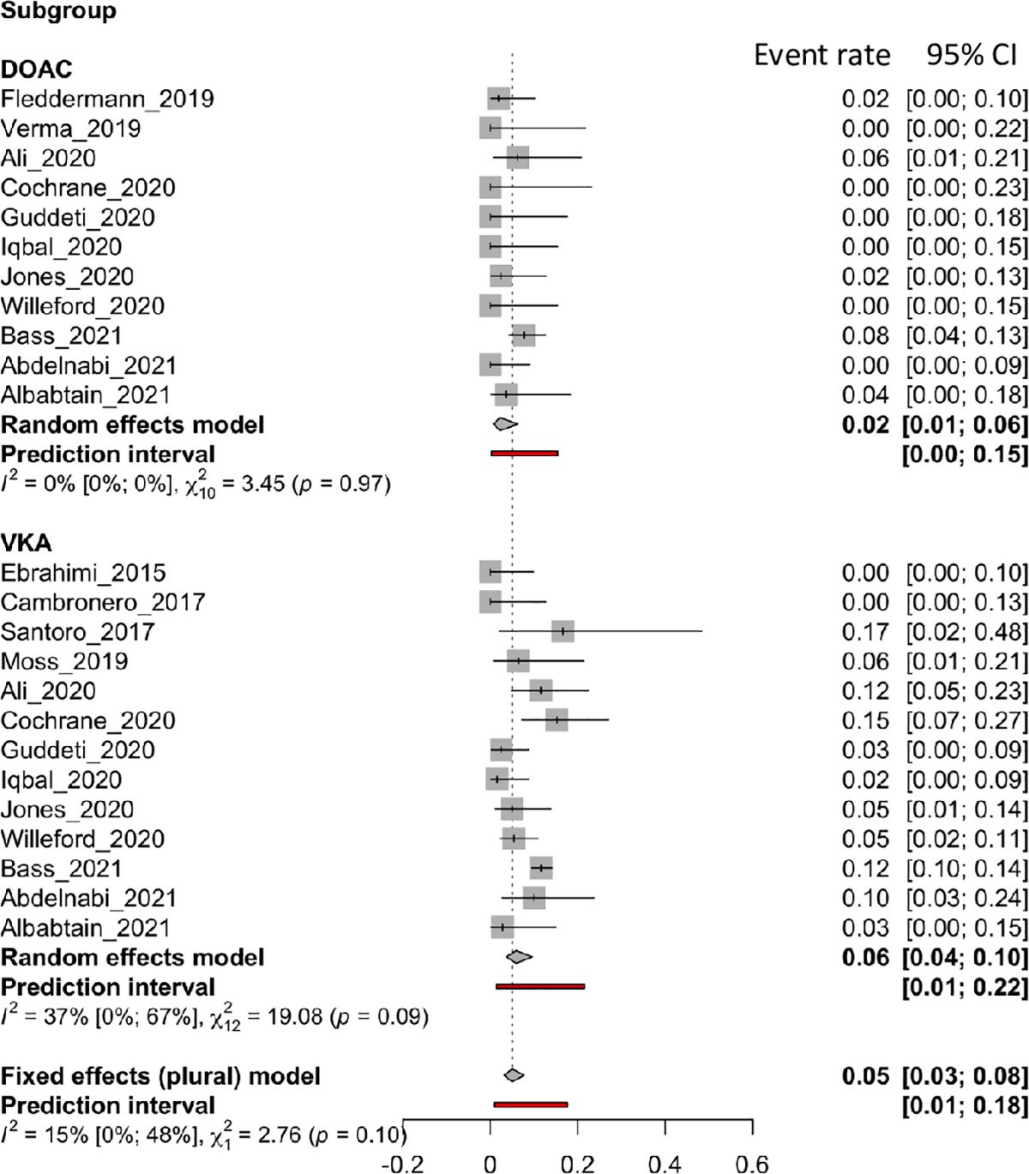
Forest plot of the proportion of stroke rate for VKA and DOAC therapy.

Fifteen articles reported an incidence of *stroke*, 13 of which were in the VKA group and 11 in the DOAC group. Fig 3 shows forest plots of the *stroke* rate. Subgroup results showed no differences between the two compound classes (VKAs: 0.06 [0.04 to 0.10]; DOACs: 0.02 [0.01 to 0.06], p = 0.0965). Moderate heterogeneity was observed in the VKAs group (I^2^: 37%), but the DOACs group showed no heterogeneity (I^2^: 0%).

### Any thromboembolism

**Fig 4:**
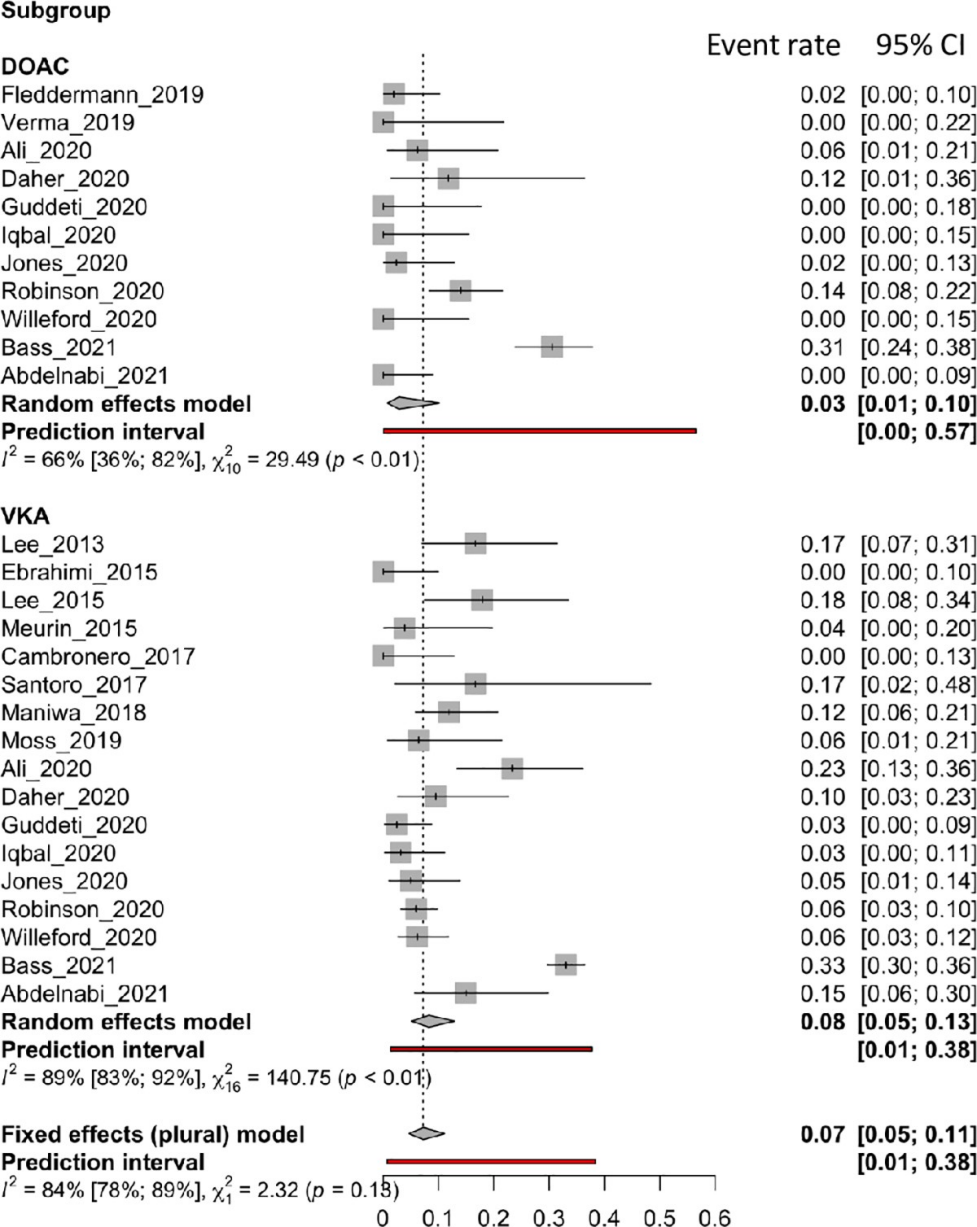
Forest plot of the proportion of any thromboembolism rate for VKA and DOAC therapy.

Nineteen articles reported *any embolism*, 17 of which were in the VKA group and 11 in the DOAC group. Fig 4 shows forest plots of the *any embolism* rate. Subgroup analysis showed no differences between the two compound classes (VKAs: 0.08 [0.05 to 0.13]; DOACs: 0.03 [0.01 to 0.10], p = 0.1278), but there was significant heterogeneity in both groups (VKAs, I^2^: 89%; DOACs, I^2^: 66%).

### Major bleeding

**Fig 5:**
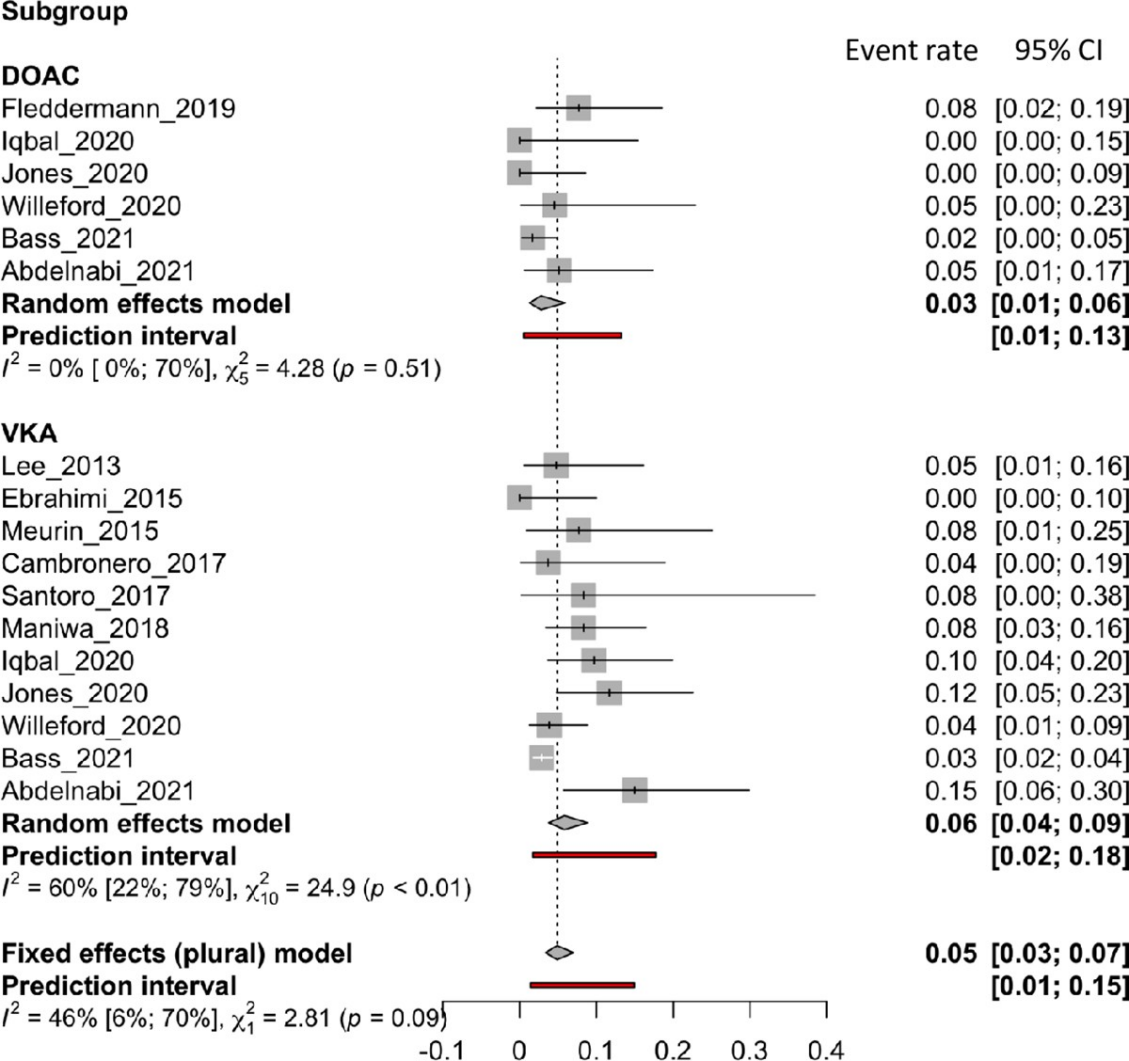
Forest plot of the proportion of major bleeding rate for VKA and DOAC therapy.

*Major bleeding* was reported in 12 articles, including 11 for VKAs and 6 for DOACs. Fig 5 shows forest plots of the *major bleeding* rate. VKA treatment strategies did not differ from DOAC strategies in the incidence of *major bleeding* (VKAs: 0.06 [0.04 to 0.09]; DOACs: 0.03 [0.01 to 0.06], p = 0.0939). There was moderate heterogeneity in the VKAs group (I^2^: 60%), but the DOACs group showed no heterogeneity (I^2^: 0%).

### Any bleeding

**Fig 6:**
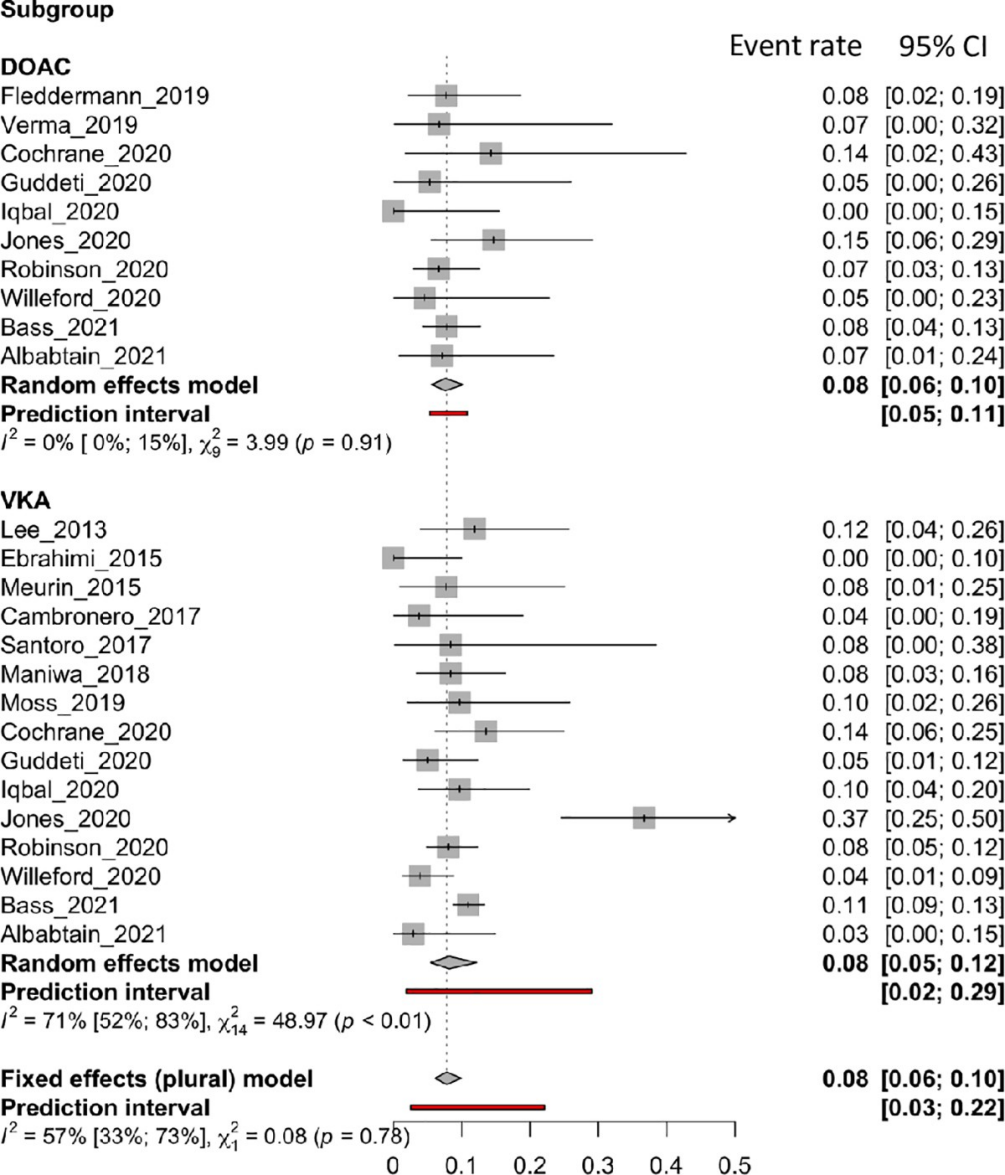
Forest plot of the proportion of any bleeding rate for VKA and DOAC therapy.

*Any bleeding* was reported in 17 articles, 15 of which were in the VKA group and 10 in the DOACs group. Fig 6 shows forest plots of the *any bleeding* rate. Subgroup results showed that incidence of *any bleeding* was similar between the two strategies (VKAs: 0.08 [0.05 to 0.12]; DOACs: 0.08 [0.06 to 0.10], p = 0.7792). Although the DOACs group had no heterogeneity (I^2^: 0%), the VKAs group had substantial heterogeneity (I^2^: 71%).

### All-cause death

**Fig 7:**
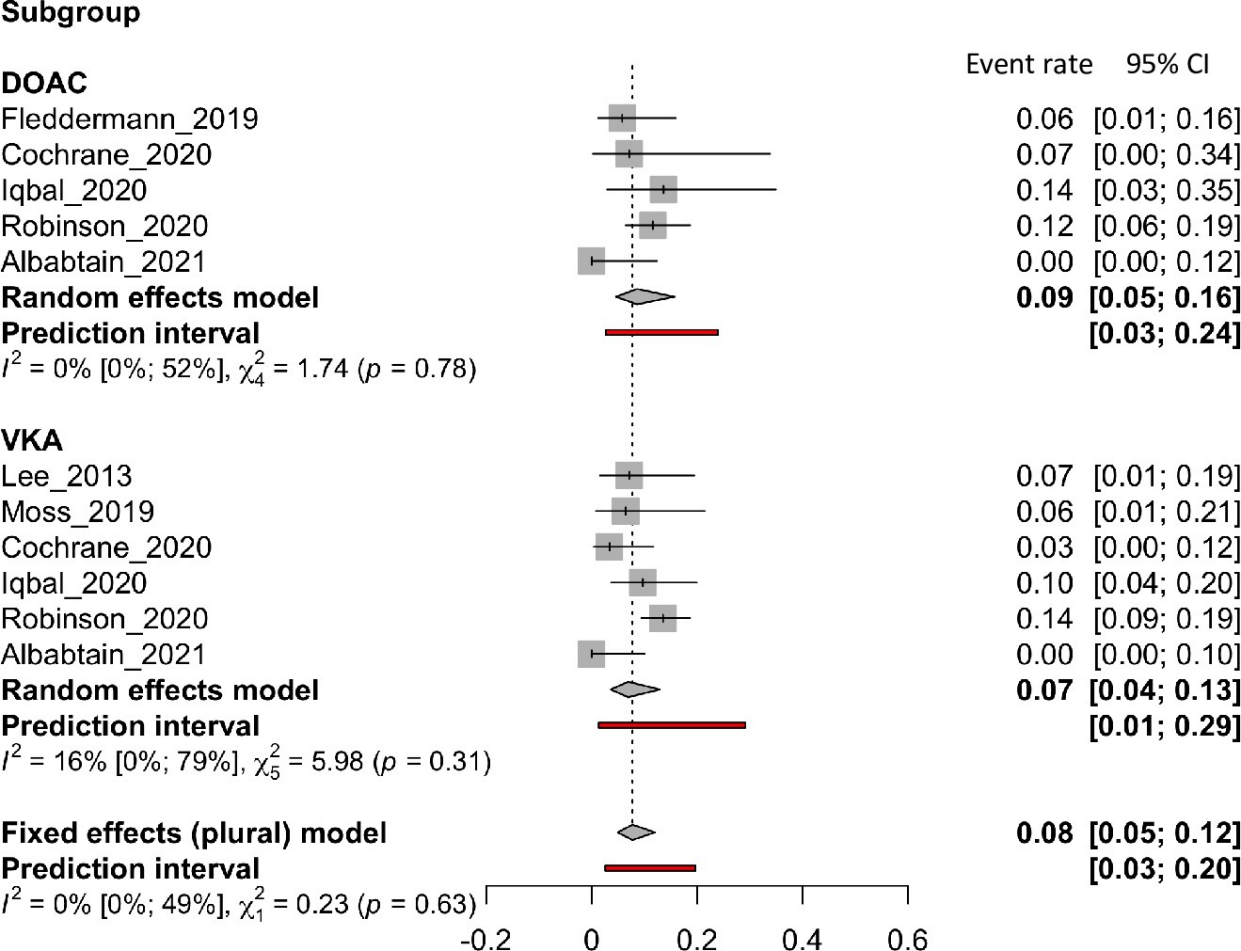
Forest plot of the proportion of all-cause death rate for VKA and DOAC therapy.

*All-cause death* was reported in 7 articles, involving 6 VKA articles and 5 DOAC articles. Fig 7 presents forest plots of the incidence of *all-cause death*. There was no difference in all-cause death between the two groups (VKAs: 0.07 [0.04 to 0.13]; DOACs: 0.09 [0.05 to 0.16], p = 0.6318). Neither group had heterogeneity (VKAs, I^2^: 16%; DOACs, I^2^: 0%).

### Meta-regression analysis

Meta-regression analysis was performed to examine the effect of follow-up duration on treatment outcomes. Duration of follow-up was not associated with any of the six events.

When meta-regression analysis was performed separately for VKAs and DOACs, there was no significant association with follow-up duration for any of the outcomes with DOACs, while with VKAs, the rate of *stroke* (estimate: -0.040, p = 0.0495) decreased significantly with longer follow-up.

### Sensitivity analysis

Sensitivity analyses were conducted to assess the robustness of the results by excluding studies with outliers. For LV *thrombus resolution*, although data from the VKA arm of Willeford et al. was excluded due to outliers, the results were unchanged from the original results (VKAs: 0.76 [0.70 to 0.80]; DOACs: 0.75 [0.68 to 0.82], p = 0.9499). For *stroke*, the results were similar to the original results, even though VKA arms of Cochrane et al. and Bass et al. were removed because they were outliers (VKAs: 0.05 [0.03 to 0.08]; DOACs: 0.02 [0.01 to 0.06], p = 0.1988). For *any embolism*, the VKA arm of Ali et al. and both arms of Bass et al. were excluded because of outliers; however, the results were the same as the original results (VKAs: 0.07 [0.05 to 0.10]; DOACs: 0.02 [0.01 to 0.08], p = 0.0988). For *any bleeding*, results after exclusion of the VKA arm of Jones et al. were similar to the original results (VKAs: 0.08 [0.06 to 0.11]; DOACs: 0.08 [0.06 to 0.10], p = 0.7625). For *major bleeding* and *all-cause death*, there were no studies with outliers.

### Publication bias

**Fig 8:**
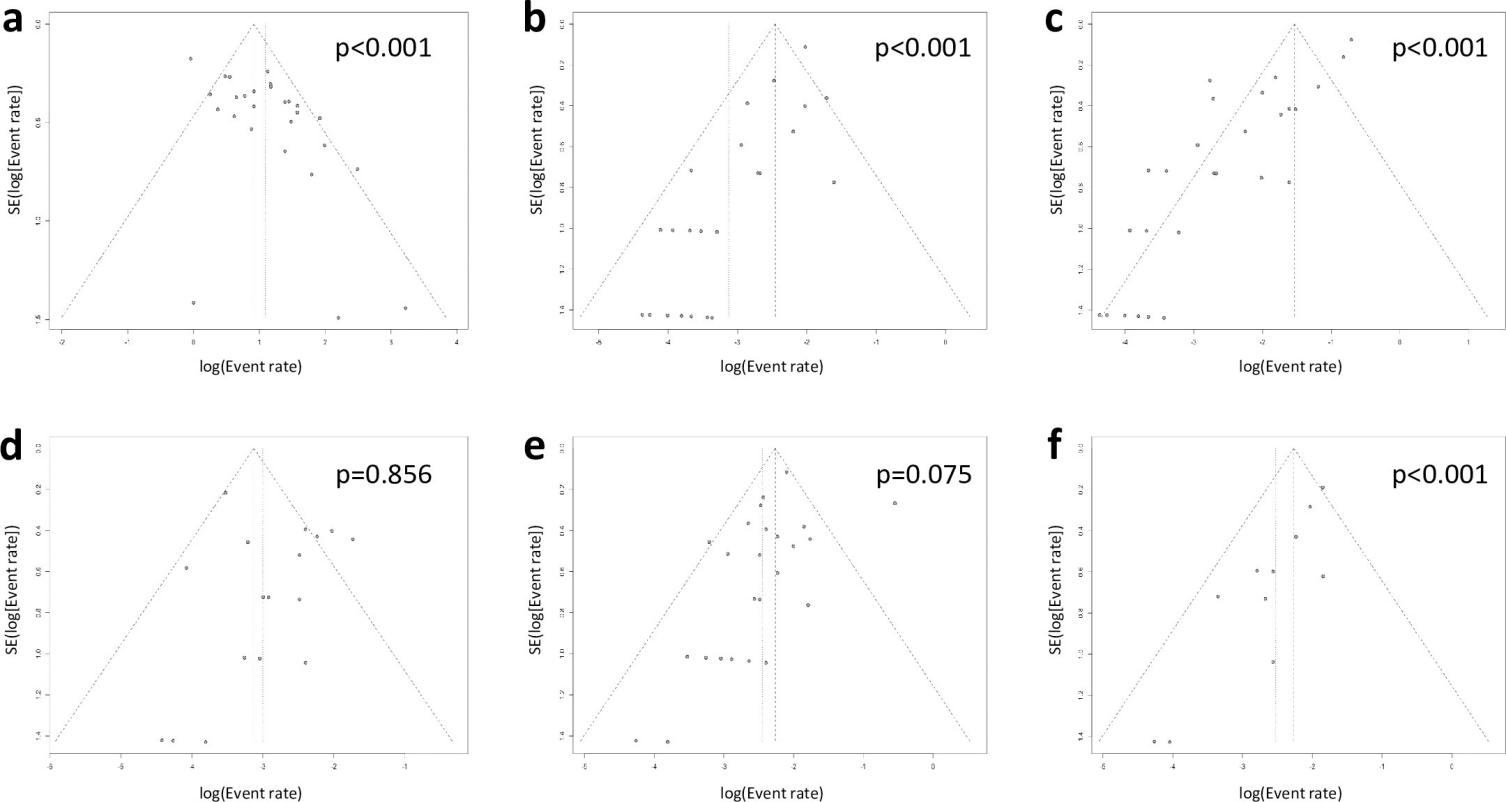
Funnel plots for (a) thrombus resolution, (b) stroke, (c) any thromboembolism, (d) major bleeding, (e) any bleeding, and (f) all-cause death. SE, standard error.

Publication bias was assessed using funnel plots (Fig 8). Egger’s test indicated significant publication bias for LV *thrombus resolution* (p<0.001), *stroke* (p<0.001), *any thromboembolism* (p<0.001), *any bleeding* (p = 0.075), and *all-cause death* (p<0.001). The pooled proportion of overall subjects adjusted using the trim and fill method was 0.67 for *thrombus resolution*, 0.09 for *stroke*, 0.21 for *any thromboembolism*, 0.06 for *major bleeding*, 0.11 for *any bleeding*, and 0.12 for *all-cause death*.

### Quality assessment

S3 Table shows study quality assessment using NOS. Most studies were of good or fair quality.

## Discussion

The main findings in this study can be summarized as follows: (1) incidence of LV *thrombus resolution*, *stroke*, *any thromboembolism*, *major bleeding*, *any bleeding*, or *all-cause death* were similar for VKAs and DOACs; (2) study heterogeneity was more often observed in the VKA arm; (3) there was no significant association between follow-up duration and any treatment outcomes with DOACs, while with VKAs, there was a significant negative correlation with follow-up duration for rates of *stroke*; (4) publication bias was found in LV *thrombus resolution*, *stroke*, *any thromboembolism*, *any bleeding*, and *all-cause death*; (5) trim and fill methods revealed that the incidence of LV *thrombus resolution* decreased and the incidence of *stroke*, *any thromboembolism*, *any bleeding*, and *all-cause death* increased after adjusting for publication bias.

### Previous studies

DOACs have many advantages over VKAs and have attracted considerable attention worldwide because they have shown efficacy and safety comparable to those of VKAs in various thrombotic diseases. In recent years, the number of DOAC-related publications has increased dramatically, and articles reporting the efficacy and safety of DOACs in treatment of LVT are becoming increasingly common. To the best of our knowledge, six systematic reviews and meta-analyses comparing VKAs and DOACs for the treatment of LVT have been published in the last several years [17–22]. Table 2 summarizes characteristics and main findings of previous meta-analyses and our study. Three studies conducted meta-analyses of five observational studies that directly compared VKAs and DOACs. In three meta-analyses, there was no significant difference in the incidence of LV *thrombus resolution* or *any thromboembolism* between the VKA and DOAC groups. Only Camilli et al. found a significantly higher incidence of bleeding events in the DOAC group. The remaining three studies performed a meta-analysis of observational studies, including articles in abstract form, that directly compared VKAs and DOACs. According to these results, there were no significant differences in LV *thrombus resolution*, *stroke*, *any thromboembolism*, *major bleeding*, or *any bleeding* between VKAs and DOACs.

**Table 2:** Main findings from previous meta-analyses and the current study.

Author/Year	Manuscript/ Abstract	Type	LVT resolution	Stroke	Any thromboembolism	Major bleeding	Any bleeding	All-cause death
Al-abcha/2020	5/0	VKA	64%	NA	5%	8%	NA	NA
		DOAC	59%	NA	9%	4%	NA	NA
		OR	0.97 (0.57–1.65)	NA	1.86 (0.98–3.50)	0.62 (0.27–1.44)	NA	NA
Camilli/2020	5/0	VKA	64%	NA	5%	NA	12%	NA
		DOAC	59%	NA	9%	NA	7%	NA
		OR	0.75 (0.53–1.05)	NA	1.86 (0.98–3.50)	NA	0.54 (0.29–0.99)	NA
Gue/2020	5/5	VKA	74%	NA	11%	6%	9%	NA
		DOAC	70%	NA	10%	4%	9%	NA
		OR	1.02 (0.56–1.86)	NA	0.90 (0.58–1.4)	0.86 (0.22–3.4)	0.93 (0.55–1.56)	NA
Saleiro/2020	5/0	VKA	55%	NA	5%	5%	NA	NA
		DOAC	44%	NA	7%	3%	NA	NA
		OR	0.91 (0.47–1.75)	NA	1.59 (0.85–2.97)	0.66 (0.31–1.4)	NA	NA
Trongtorsak/ 2020	6/2	VKA	NA	NA	NA	NA	NA	NA
		DOAC	NA	NA	NA	NA	NA	NA
		OR	1.09 (0.94–1.27)	NA	1.12 (0.91–1.39)	NA	0.94 (0.59–1.51)	NA
Michael/2021	11/7	VKA	70%	11%	22%	NA	10%	13%
		DOAC	69%	6%	17%	NA	7%	13%
		OR	1.29 (0.83–1.99)	0.63 (0.42–0.96)	0.83 (0.53–1.33)	NA	0.72 (0.50–1.02)	1.01 (0.64–1.57)
Saleh/2021	8/5	VKA	72%	NA	21%	8%	NA	NA
		DOAC	71%	NA	16%	7%	NA	NA
		OR	1.22 (0.79–1.88)	NA	0.88 (0.56–1.38)	0.87 (0.55–1.39)	NA	NA
Zhou/2021	5/4	VKA	79%	11%	24%	9%	NA	NA
		DOAC	83%	8%	25%	9%	NA	NA
		OR	1.34 (0.62–2.9)	0.79 (0.50–1.23)	1.50 (0.76–2.97)	0.82 (0.47–1.42)	NA	NA
Our study	20/0	VKA	75%	5%	7%	5%	8%	8%
		DOAC	75%	2%	3%	3%	8%	9%

DOAC, direct oral anticoagulant; LVT, left ventricular thrombosis; NA, not applicable; OR, odds ratio; RR, relative risk; VKA, vitamin k antagonist.

Previous meta-analyses included only direct comparisons of VKAs and DOACs, and some also included abstracts. However, there are a number of published papers that report outcomes for either VKAs or DOACs in treatment of LVT, and these articles can be included in the meta-analysis to reduce selection bias and to generalize results [29–36, 38, 39].

### Current study

Our meta-analysis included a higher number of articles (23) and subjects (2,612) than previous meta-analyses. Incidence of LV *thrombus resolution*, *stroke*, *any thromboembolism*, *major bleeding*, or *any bleeding* were similar for VKAs and DOACs, which agreed with previous results. Although DOACs showed no heterogeneity for *stroke*, *major bleeding*, *any bleeding* or *all-cause death*, VKAs had heterogeneity, except for *all-cause death*. Our results showed no significant differences in *all-cause death* between the two treatment groups. Meta-regression analysis revealed that the rate of *stroke* significantly decreased with longer follow-up duration in VKAs. However, there were no significant associations with follow-up duration for any of the outcomes with DOACs. The lack of differences in safety or efficacy of DOACs, depending on the follow-up duration, makes long-term DOAC treatment useful for patients with LVT.

Sensitivity analysis yielded similar results when outliers for LV *thrombus resolution*, *stroke*, *any embolism*, and *any bleeding* were removed. Regarding major bleeding and all-cause death, no studies had outliers. These results support robustness of the present results.

Publication bias was also found in the majority of comparisons. Many negative studies tend not to be published, and this may distort the results of meta-analyses. In order to overcome publication bias, a trim-and-fill method was used to add presumed missing studies, which resulted in lower LV *thrombus resolution* rates and higher rates of *stroke*, *any thromboembolism*, *any bleeding*, and *all-cause death*. Thus, prospective randomized controlled studies are required to fill the gap between observed incidence from meta-analysis and predicted incidence.

### Study limitations

This study had several limitations. First, follow-up duration for determining outcomes varied considerably among studies, which could affect observed results. Second, all but one study in this meta-analysis included non-randomized observational studies, which are prone to bias. However, the quality of each study was assessed using NOS, and the quality of most studies was high. Third, the definition of *major bleeding* differed among the studies included. This may have caused bias because studies employed different criteria for bleeding, such as BARC, GUSTO, and TIMI. Fourth, because our analysis was based on data summarized by each author and individual patient data were not available, our results should be interpreted with some caution. Fifth, although clinical benefit increases with longer time in therapeutic range (TTR) and because adverse events increase with shorter TTR, the relationship between TTR and efficacy and safety of VKA treatment was not examined, which may have influenced our results. Sixth, trial sequential analysis could not be conducted because some data were single-arm studies. Finally, some patients were prescribed antiplatelet drugs such as aspirin, clopidogrel, and prasugrel, which may have affected our results.

## Conclusions

Therapeutic efficacy and side effects of DOACs in treatment of patients with LVT were quite similar to those of VKAs regarding LV *thrombus resolution*, systemic *embolization*, *bleeding*, or *all-cause death*. Our results highlight the need for the RCT to determine an optimal anticoagulant strategy for treatment of LVT.

## Supporting information

S1 Checklist PRISMA 2009 checklist. (DOC)

S1 Table. Search strategies (PDF)

S2 Table Clinical characteristics of study subjects. (PDF)

S3 Table Study quality assessment using the Newcastle Ottawa Scale. (PDF)

S1 File (PDF)

S1 Data (XLSX)

S2 Data (XLSX)

S3 Data (XLSX)

S4 Data (XLSX)

S5 Data (XLSX)

S6 Data (XLSX)
