# Supplementary material for: Therapeutic efficacy of direct oral anticoagulants and vitamin K antagonists for left ventricular thrombus: Systematic review and meta-analysis
Source: PLoS One. 2021 Jul 26;16(7):e0255280. doi: 10.1371/journal.pone.0255280 (PMC8312978; doi:10.1371/journal.pone.0255280)
Supplement: S1 Table. Search strategies — (PDF) [file pone.0255280.s002.pdf]

S1 Table: Search strategies.

Cochrane library Search LVT for VKA or DOAC
Search date on 28/ May/ 2021

I. **Cochrane library:**
   1: (anticoagulant OR (vitamin k antagonist) OR warfarin): ti, ab, kw
   2: ((left ventricular) OR (left ventricle) OR intraventricular): ti, ab, kw
   3: (thrombus OR thrombi): ti, ab, kw
   3: #1 AND #2 AND #3
   Results: 104 articles

Scopus Search LVT for VKA or DOAC
Search date on 28/May/2021

II. **Scopus:**
   1: TITLE-ABS-KEY (anticoagulant OR (vitamin AND k AND antagonist) OR warfarin)
   2: TITLE-ABS-KEY ((left AND ventricular) OR (left AND ventricle) OR intraventricular)
   3: TITLE-ABS-KEY (thrombus OR thrombi)
   4: #1 AND #2 AND #3
   Results: 1,946 articles

PubMed Search LVT for VKA or DOAC
Search date on 28/May/2021

III. **PubMed:**
   Keywords: Search (anticoagulant OR (vitamin k antagonist) OR warfarin) AND ((left ventricular) OR (left ventricle) OR intraventricular) AND (thrombus OR thrombi)
   Results: 1,988 articles

Records After Duplicates Removed n=3034
