# Supplementary material for: Therapeutic efficacy of direct oral anticoagulants and vitamin K antagonists for left ventricular thrombus: Systematic review and meta-analysis
Source: PLoS One. 2021 Jul 26;16(7):e0255280. doi: 10.1371/journal.pone.0255280 (PMC8312978; doi:10.1371/journal.pone.0255280)
Supplement: S2 Table — (PDF) [file pone.0255280.s003.pdf]

S2 Table: Clinical characteristics of study subjects.

VKA

Author	Number	Age	Male	HT	DM	DL	CKD	CAD	AFib	Smoking
Lee_2013	42	64 ± 14	33	24	11	27	4	17	2	13
Ebrahimi_2015	35	NA	31	16	13	15	NA	NA	NA	12
Lee_2015	39	NA	NA	NA	NA	NA	NA	NA	NA	NA
Meurin_2015	26	57 ± 13	20	7	3	5	NA	NA	1	NA
Cambronero_2017	27	58 ± 13	23	10	6	12	NA	NA	NA	16
Santoro_2017	12	72 ± 10	0	11	3	7	NA	NA	NA	3
Maniwa_2018	84	62 ± 12	74	52	38	53	NA	3	6	51
McCarthy_2019	94	NA	NA	NA	NA	NA	NA	NA	NA	NA
Moss_2019	31	NA	NA	NA	NA	NA	NA	31	NA	NA
Ali_2020	60	58 ± 16	49	NA	18	NA	NA	NA	18	7
Cochrane_2020	59	NA	45	NA	23	NA	22	36	NA	NA
Daher_2020	42	61 ± 13	35	17	9	18	NA	NA	NA	25
Guddeti_2020	80	61 ± 12	55	61	34	NA	NA	53	18	NA
Iqbal_2020	62	62 ± 14	55	18	19	9	NA	NA	3	28
Jones_2020	60	61 ± 14	51	22	10	19	5	NA	NA	20
Ratnayake_2020	42	NA	NA	NA	NA	NA	NA	NA	NA	NA
Robinson_2020	236	58 ± 15	170	177	92	125	NA	NA	45	NA
Willeford_2020	129	NA	104	54	37	NA	19	68	24	NA
Bass_2021	769	62 ± 15	545	NA	NA	NA	268	443	352	NA
Abdelnabi_2021	40	50 ± 13	24	19	19	16	NA	NA	NA	22
Albabtain_2021	35	59 ± 16	34	19	16	NA	NA	NA	2	NA
	2004	61 ± 15 (n=1575)	75% (1348/1798)	56% (507/910)	34% (351/1029)	46% (306/666)	30% (318/1059)	55% (651/1194)	31% (471/1523)	42% (197/464)

DOAC

Author	Number	Age	Male	HT	DM	DL	CKD	CAD	AFib	Smoking
Fleddermann_2019	52	64 ± 14	37	NA	NA	NA	NA	NA	NA	NA
McCarthy_2019	4	NA	NA	NA	NA	NA	NA	NA	NA	NA
Verma_2019	15	41 ± 19	9	NA	NA	NA	NA	NA	3	NA
Ali_2020	32	59 ± 12	26	NA	12	NA	NA	NA	9	7
Cochrane_2020	14	NA	11	NA	6	NA	5	7	NA	NA
Daher_2020	17	57 ± 14	14	10	2	5	NA	NA	NA	10
Guddeti_2020	19	61 ± 13	15	15	3	NA	NA	11	4	NA
Iqbal_2020	22	62 ± 13	20	9	19	4	NA	NA	3	10
Jones_2020	41	59 ± 14	33	23	7	19	5	NA	NA	8
Ratnayake_2020	2	NA	NA	NA	NA	NA	NA	NA	NA	NA
Robinson_2020	121	58 ± 15	94	86	36	71	NA	NA	30	NA
Willeford_2020	22	NA	17	8	4	NA	2	15	3	NA
Bass_2021	180	63 ± 17	125	NA	NA	NA	53	77	111	NA
Abdelnabi_2021	39	49 ± 12	21	23	23	22	NA	NA	NA	20
Albabtain_2021	28	58 ± 18	24	13	12	NA	NA	NA	1	NA
	608	59 ± 16 (n=566)	74% (446/602)	61% (187/309)	35% (124/355)	50% (121/240)	25% (65/257)	47% (110/235)	37% (164/439)	36% (55/151)

Data are expressed as number or means ± standard deviations.
AFib, atrial fibrillation; CAD, coronary artery disease; CKD, chronic kidney disease; DL, dyslipidemia; DM, diabetes mellitus; DOAC, direct oral anticoagulant; HT, hypertension; NA, not applicable; VKA, vitamin k antagonist.
