# Supplementary material for: Therapeutic efficacy of direct oral anticoagulants and vitamin K antagonists for left ventricular thrombus: Systematic review and meta-analysis
Source: PLoS One. 2021 Jul 26;16(7):e0255280. doi: 10.1371/journal.pone.0255280 (PMC8312978; doi:10.1371/journal.pone.0255280)
Supplement: S3 Table — (PDF) [file pone.0255280.s004.pdf]

S3 Table: Study quality assessment using the Newcastle Ottawa Scale.

Author	Selection				Comparability	Outcome			Total
	Representativeness	Non-exposed	Ascertainment	Demonstration of outcome		Assessment	Follow-up long enough	Adequacy of follow-up	
Lee	★	★	★	★	★★	★	★	★	9
Ebrahimi	Descriptive study								-
Lee	★	★	★	★	★★	★	★	★	9
Meurin	★	★	★	★	-	★	★	★	7
Cambronero	★	★	★	★	★	★	★	★	8
Santoro	★	★	★	★	★	★	★	★	8
Maniwa	★	★	★	★	★	★	★	★	8
Fleddermann	★	★	★	★	-	★	★	★	7
McCarthy	★	★	★	★	-	★	★	★	7
Moss	★	★	★	★	★	★	★	★	8
Verma	Descriptive study								-
Ali	★	★	★	★	★	★	★	-	7
Cochrane	★	★	★	★	★★	★	★	★	9
Daher	★	★	★	★	★★	★	★	★	9
Guddeti	★	★	★	★	★	★	★	★	8
Iqbal	★	★	★	★	★	★	★	★	8
Jones	★	★	★	★	★★	★	★	★	9
Ratnayake	★	★	★	★	-	★	★	★	7
Robinson	★	★	★	★	★★	★	★	-	8
Willeford	★	★	★	★	★★	★	★	★	9
Bass	★	★	★	★	★★	★	★	★	9
Abdelnabi	Randomized controlled trial								-
Albabtain	★	★	★	★	★	★	★	★	8
