# Supplementary material for: Therapeutic efficacy of direct oral anticoagulants and vitamin K antagonists for left ventricular thrombus: Systematic review and meta-analysis
Source: PLoS One. 2021 Jul 26;16(7):e0255280. doi: 10.1371/journal.pone.0255280 (PMC8312978; doi:10.1371/journal.pone.0255280)

LV thrombus resolution

Study	DOAC Events	DOAC Total	VKA Events	VKA Total	OR	95%-CI	Weight (fixed)	Weight (random)
McCarthy_2019	4	4	71	94	2.96	[0.15; 57.01]	1.4%	1.6%
Ali_2020	18	32	37	60	0.80	[0.33; 1.91]	21.7%	18.4%
Cochrane_2020	12	14	45	59	1.87	[0.37; 9.36]	4.8%	5.4%
Daher_2020	12	17	30	42	0.96	[0.28; 3.32]	9.8%	9.1%
Iqbal_2020	13	20	42	55	0.57	[0.19; 1.74]	15.1%	11.3%
Jones_2020	34	41	38	60	2.81	[1.07; 7.40]	10.2%	14.9%
Ratnayake_2020	1	2	34	42	0.24	[0.01; 4.18]	3.0%	1.7%
Willeford_2020	13	22	63	129	1.51	[0.60; 3.79]	14.5%	16.6%
Abdelnabi_2021	34	39	32	40	1.70	[0.50; 5.74]	7.8%	9.4%
Albabtain_2021	20	28	24	35	1.15	[0.39; 3.40]	11.8%	11.8%
**Fixed effect model**		**219**		**616**	**1.26**	**[0.88; 1.82]**	**100.0%**	**--**
**Random effects model**					**1.23**	**[0.85; 1.79]**	**--**	**100.0%**

Heterogeneity: $I^2 = 0\%$, $\tau^2 = 0$, $p = 0.53$

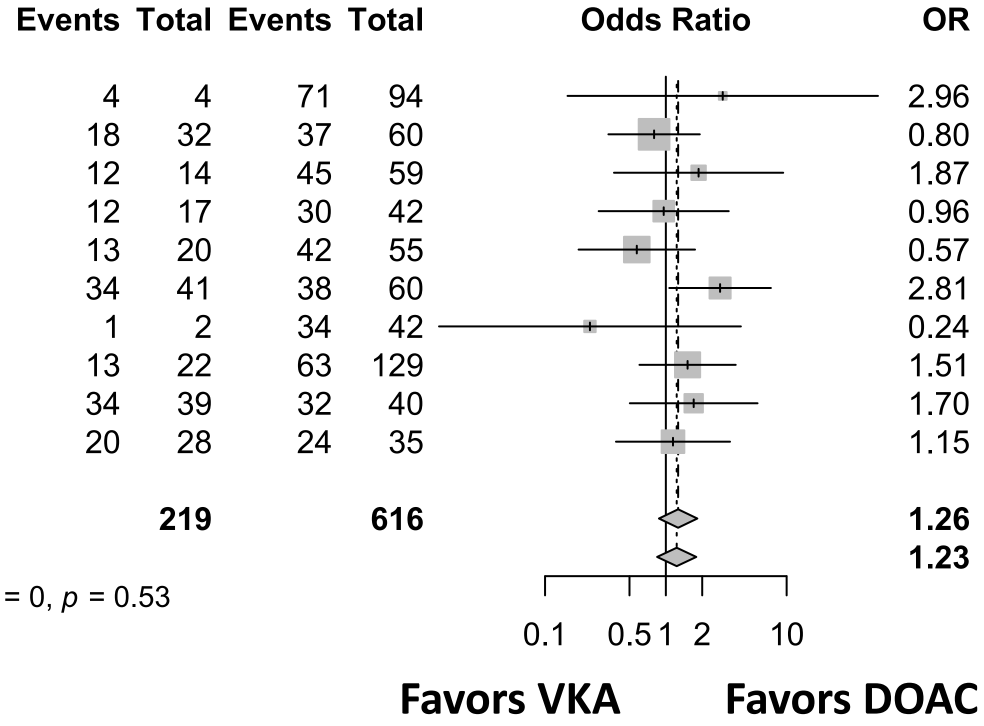

Stroke

Study	DOAC Events	DOAC Total	VKA Events	VKA Total	OR	95%-CI	Weight (fixed)	Weight (random)
Ali_2020	2	32	7	60	0.50	[0.10; 2.59]	8.9%	9.1%
Cochrane_2020	0	14	9	59	0.18	[0.01; 3.34]	7.2%	2.9%
Guddeti_2020	0	19	2	80	0.81	[0.04; 17.46]	1.9%	2.6%
Iqbal_2020	0	22	1	62	0.91	[0.04; 23.19]	1.5%	2.3%
Jones_2020	1	41	3	60	0.48	[0.05; 4.73]	4.6%	4.6%
Willeford_2020	0	22	7	129	0.36	[0.02; 6.58]	4.3%	2.9%
Bass_2021	14	180	90	769	0.64	[0.35; 1.15]	61.4%	69.9%
Abdelnabi_2021	0	39	4	40	0.10	[0.01; 1.97]	8.6%	2.8%
Albabtain_2021	1	28	1	35	1.26	[0.08; 21.07]	1.7%	3.0%
**Fixed effect model**		**397**		**1294**	**0.55**	**[0.34; 0.89]**	**100.0%**	**--**
**Random effects model**					**0.57**	**[0.35; 0.94]**	**--**	**100.0%**

Heterogeneity: $I^2 = 0\%$, $\tau^2 = 0$, $p = 0.96$

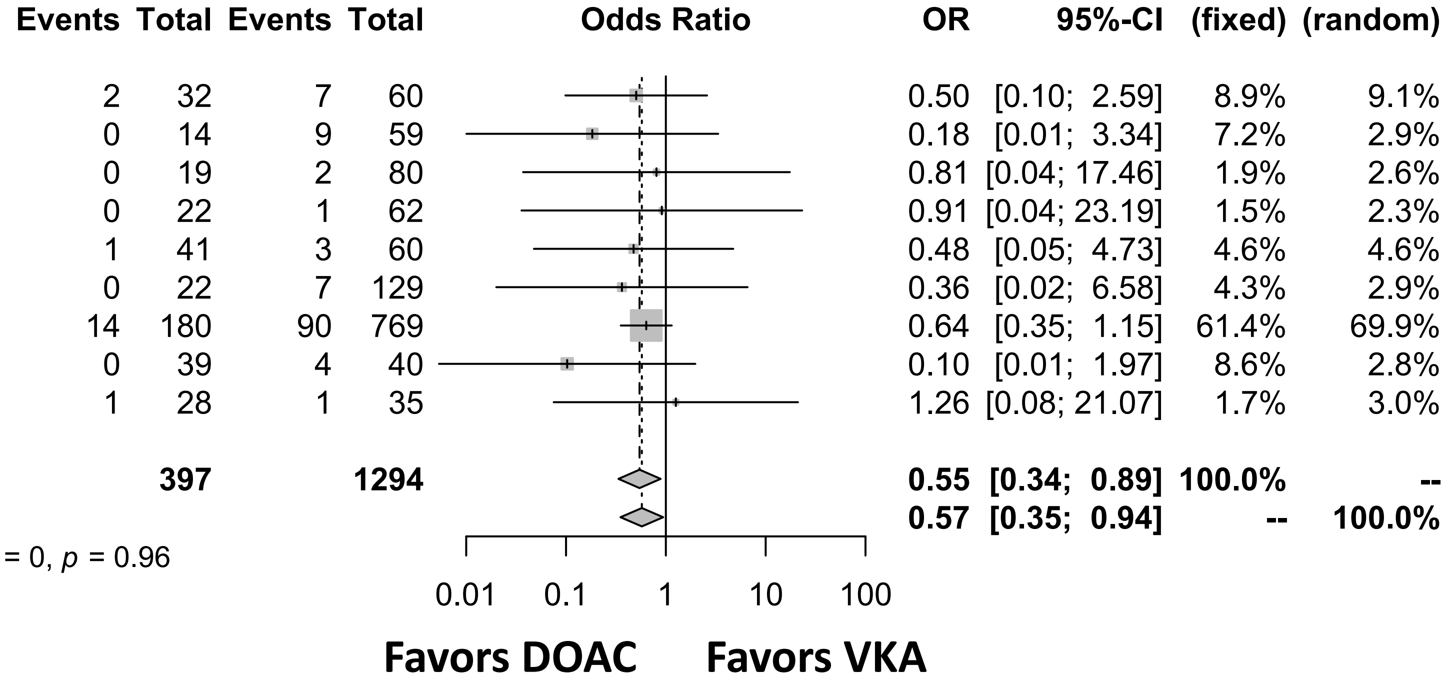

Any thromboembolism

Study	DOAC Events	DOAC Total	VKA Events	VKA Total	OR	95%-CI	Weight (fixed)	Weight (random)
Ali_2020	2	32	14	60	0.22	[0.05; 1.03]	9.2%	12.0%
Daher_2020	2	17	4	42	1.27	[0.21; 7.66]	2.0%	9.8%
Guddeti_2020	0	19	2	80	0.81	[0.04; 17.46]	1.0%	4.2%
Iqbal_2020	0	22	2	62	0.54	[0.02; 11.64]	1.3%	4.2%
Jones_2020	1	41	3	60	0.48	[0.05; 4.73]	2.4%	6.8%
Robinson_2020	17	121	14	236	2.59	[1.23; 5.46]	8.2%	23.5%
Willeford_2020	0	22	8	129	0.32	[0.02; 5.70]	2.5%	4.7%
Bass_2021	55	180	254	769	0.89	[0.63; 1.27]	67.1%	30.2%
Abdelnabi_2021	0	39	6	40	0.07	[0.00; 1.24]	6.4%	4.6%
**Fixed effect model**		**493**		**1478**	**0.89**	**[0.67; 1.19]**	**100.0%**	**--**
**Random effects model**					**0.79**	**[0.40; 1.55]**	**--**	**100.0%**

Heterogeneity: $I^2 = 47\%$, $\tau^2 = 0.3592$, $p = 0.06$

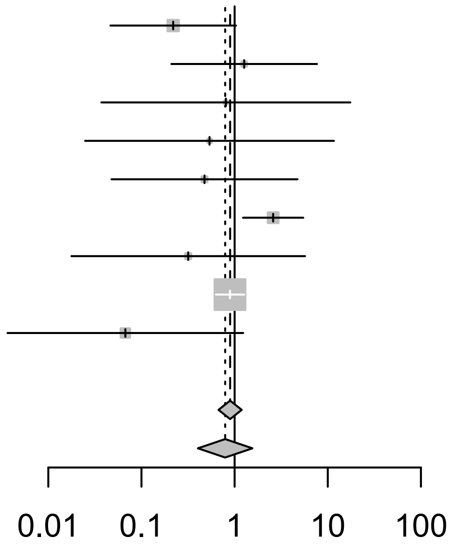

Major bleeding

Study	DOAC Events	DOAC Total	VKA Events	VKA Total	OR	95%-CI	Weight (fixed)	Weight (random)
Iqbal_2020	0	22	6	62	0.19	[0.01; 3.57]	13.8%	7.9%
Jones_2020	0	41	7	60	0.09	[0.00; 1.55]	24.5%	8.1%
Willeford_2020	1	22	5	129	1.18	[0.13; 10.62]	5.6%	14.0%
Bass_2021	3	180	22	769	0.58	[0.17; 1.94]	33.3%	45.6%
Abdelnabi_2021	2	39	6	40	0.31	[0.06; 1.62]	22.8%	24.3%
**Fixed effect model**		**304**		**1060**	**0.38**	**[0.17; 0.85]**	**100.0%**	**--**
**Random effects model**					**0.43**	**[0.19; 0.98]**	**--**	**100.0%**

Heterogeneity: $I^2 = 0\%$, $\tau^2 = 0$, $p = 0.60$

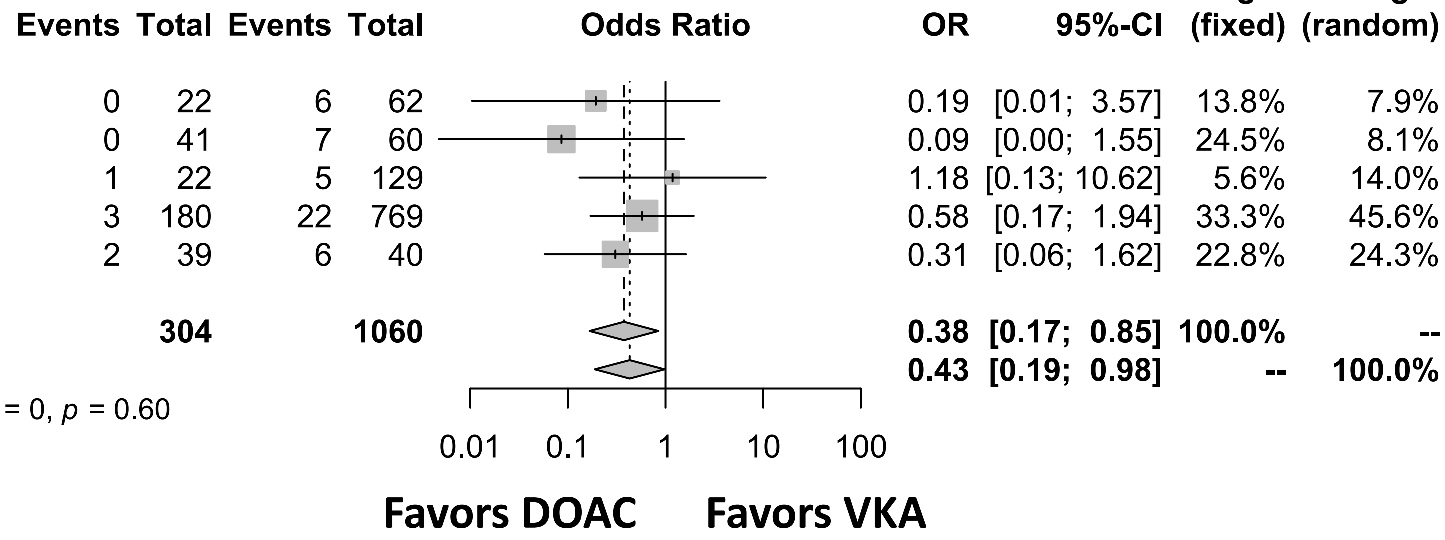

Any bleeding

Study	DOAC Events	DOAC Total	VKA Events	VKA Total	OR	95%-CI	Weight (fixed)	Weight (random)
Cochrane_2020	2	14	8	59	1.06	[0.20; 5.66]	4.0%	5.7%
Guddeti_2020	1	19	4	80	1.06	[0.11; 10.02]	2.2%	3.2%
Iqbal_2020	0	22	6	62	0.19	[0.01; 3.57]	5.1%	1.9%
Jones_2020	6	41	22	60	0.30	[0.11; 0.82]	23.0%	15.6%
Robinson_2020	8	121	19	236	0.81	[0.34; 1.90]	18.1%	21.8%
Willeford_2020	1	22	5	129	1.18	[0.13; 10.62]	2.1%	3.3%
Bass_2021	14	180	84	769	0.69	[0.38; 1.24]	44.3%	45.9%
Albabtain_2021	2	28	1	35	2.62	[0.22; 30.43]	1.2%	2.7%
**Fixed effect model**		**447**		**1430**	**0.65**	**[0.44; 0.97]**	**100.0%**	**--**
**Random effects model**					**0.67**	**[0.45; 1.00]**	**--**	**100.0%**

Heterogeneity: $I^2 = 0\%$, $\tau^2 = 0$, $p = 0.62$

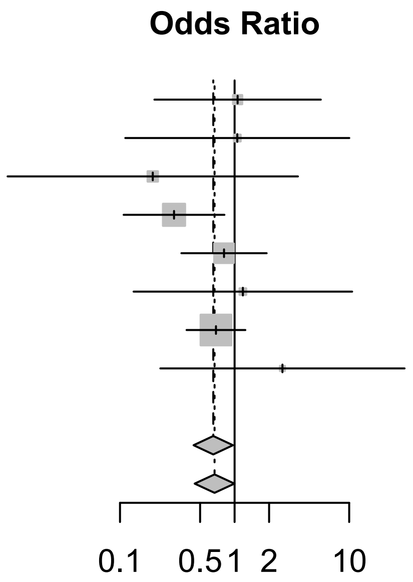

Favors DOAC **Favors VKA**

All-cause death

Study	DOAC Events	DOAC Total	VKA Events	VKA Total	OR	95%-CI	Weight (fixed)	Weight (random)
Cochrane_2020	1	14	2	59	2.19	[0.18; 26.05]	3.2%	5.7%
Iqbal_2020	3	22	6	62	1.47	[0.34; 6.48]	12.0%	16.0%
Robinson_2020	14	121	32	236	0.83	[0.43; 1.63]	84.8%	78.2%
Albabtain_2021	0	28	0	35			0.0%	0.0%
**Fixed effect model**		**185**		**392**	**0.95**	**[0.53; 1.72]**	**100.0%**	**--**
**Random effects model**					**0.97**	**[0.53; 1.75]**	**--**	**100.0%**

Heterogeneity: $I^2 = 0\%$, $\tau^2 = 0$, $p = 0.63$

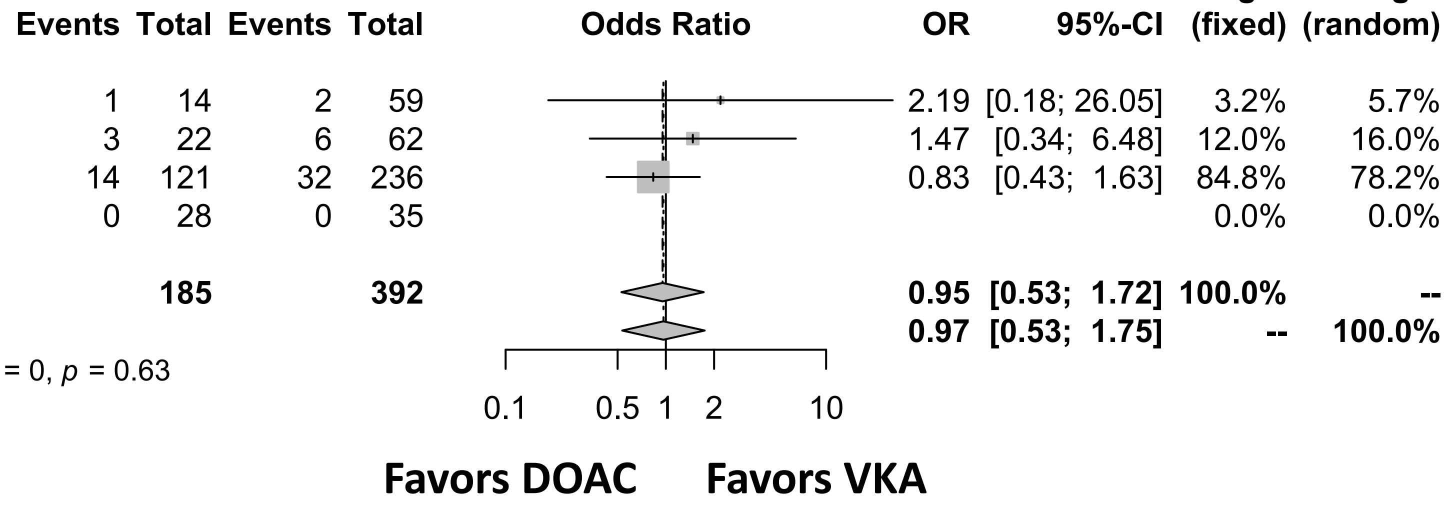

Supplement: S1 File — (PDF) [file pone.0255280.s005.pdf]
